# Snowflake cataract in Down’s syndrome

**DOI:** 10.11604/pamj.2023.44.13.37833

**Published:** 2023-01-06

**Authors:** Rasika Bagewadi, Sachin Daigavane

**Affiliations:** 1 Department of Ophthalmology, Jawaharlal Nehru Medical College, Datta Meghe Institute of Medical Sciences, Sawangi (Meghe), Wardha, Maharashtra, India

**Keywords:** Snowflake cataract, cataract, diabetes, insulin

## Image in medicine

A 20-year-old male patient came with complaints of defective vision in bilateral eyes for one year. The patient was diagnosed the case of Down's syndrome. He had type 1 diabetes and hypothyroidism for which he received insulin and levothyroxine. His best corrected visual acuity (BCVA) in right eye was 6/60 and left eye was 6/18. On anterior segment examination, greyish-white sheet-like opacities were seen in the cortical area with posterior subcapsular cataract. His intraocular pressure and other ocular examinations were normal. Posterior segment could not be evaluated due to hazy media. B-scan ocular ultrasound was done and was normal in both eyes. He was advised to do cataract extraction by phacoemulsification with intraocular lens implantation surgery.

**Figure 1:**
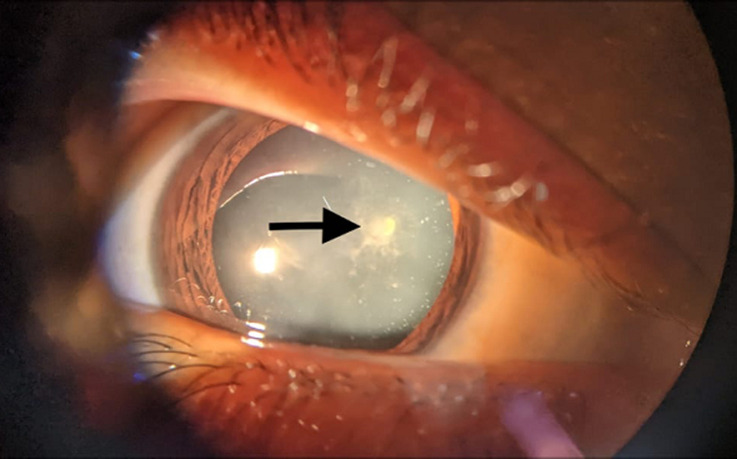
snowflake cataract with posterior subcapsular cataract (arrow marked) in right eye

